# Suboptimal health: a new health dimension for translational medicine

**DOI:** 10.1186/2001-1326-1-28

**Published:** 2012-11-14

**Authors:** Wei Wang, Yuxiang Yan

**Affiliations:** 1 School of Medical Sciences, Edith Cowan University, Perth, Australia; 2 Beijing Municipal Key Laboratory of Clinical Epidemiology and School of Public Health, Capital Medical University, Beijing, China; 3 Graduate School of the Chinese Academy of Sciences, Beijing, China

**Keywords:** Suboptimal health, Instrument SHSQ-25, Chronic disease, Translational medicine

## Abstract

**Background:**

One critical premise of disease-related biomarkers is the definition of the counterpart normality. Contrary to pre-clinical models that can be carefully tailored according to scientific need, heterogeneity and uncontrollability is the essence of humans in health studies. Fully characterization of consistent parameters that define the normal population is the basis to individual differences normalization irrelevant to a given disease process. Self claimed normal status may not represent health because asymptomatic subjects may carry chronic diseases or diseases at their early stage such as cancer, diabetes and hypertension.

**Methods:**

This paper exemplifies the characterization of the suboptimal health status (SHS) which represents a new public health problem in a population with ambiguous health complaints such as general weakness, unexplained medical syndrome and chronic fatigue. We applied clinical informatics approaches and developed a questionnaire for measuring SHS. The validity and reliability of this approach were evaluated in a small pilot study and then in a cross-sectional study of 3,405 individuals.

**Results:**

The final questionnaire congregated into a score (SHSQ-25) which could significantly distinguish among several abnormal conditions.

**Conclusion:**

SHSQ-25 could be used as a translational medicine instrument for health measuring in the general population.

## Background

Health is the level of functional or metabolic efficiency of a living being. In humans, it indicates the general condition of a people's mind, body and spirit, usually meaning to be free from illness, stress, injury or pain [1]. In 1946, the World Health Organization (WHO) defined health as "a state of complete physical, mental, and social well-being and not merely the absence of disease or infirmity" [2,3]. Although WHO health definition has been subject to controversy, in particular as having a lack of operational value and the problem created by use of the word "complete", it remains the most enduring [4,5]. Classification systems such as the WHO Family of International Classifications, including the International Classification of Functioning, Disability and Health (ICF) and the International Classification of Diseases (ICD), are commonly used to define and measure the components of health.

In China, there has been an increase in the number of people who report suboptimal health in the absence of a diagnosable condition, which we coined as suboptimal health status (SHS) [6,7]. SHS is a physical state between health and disease, and is characterized by 1) the perception of health complaints, general weakness and low energy within a period of 3 months. 2) It is regarded as a subclinical, reversible stage of chronic disease.

The existence of a reliable and valid instrument to assess SHS will be essential. Therefore, we developed and validated a comprehensive questionnaire SHSQ-25 to assess SHS among urban Chinese [6]. The SHSQ-25 accounts for the multidimensionality of SHS by encompassing the domains of 1) fatigue, 2) the cardiovascular system, 3) the digestive tract, 4) the immune system, and 5) mental status. It is short and easy to complete, and therefore suitable for use in studies of the general population.

Instrument: Sub-health Status Questionnaire (Additional file 1: SHSQ-25) [6].

An example of association study of suboptimal health status and cardiovascular risk factors in urban Chinese workers [7].

## Results and discussion

A cross-sectional study was conducted among workers in urban Beijing. A random third of all the 64 companies whose workers took annual physical examination for at least two consecutive years at the physical examination center of Beijing Xuanwu Hospital, Capital Medical University were selected for cluster sampling. Participants had to meet the following inclusion criteria: (1) no history of somatic or psychiatric abnormalities, as confirmed by their medical records, (2) age from 20 through 60 years; and (3) no history of medication consumption in the previous 2 weeks. All participants attended a standardized examination protocol in Beijing Xuanwu Hospital, including medical history, physical examination, blood hematology and biochemistry analysis, rest electrocardiography, and abdominal ultrasonography. We excluded individuals who met the diagnostic criteria of specific disease concerning cardiovascular system, respiratory system, genitourinary system, digestive system, hematic system, and diabetes. Of the 4,881 workers from 64 companies, 1,476 were excluded from the study. Finally, a total of 3,405 people were investigated in this study. SHSQ-25 self-reported questionnaire was used to assess the respondents’ sociodemographics and SHS. Median was used as a cut point in grouping into high versus low of two dimensions of SHS. To assure comparability of the findings, all participants were examined by the physicians who were specially trained for the study. Both the hospital and university research ethical committees approved the study, and written informed consents were obtained from all participants.

SHS was measured by the suboptimal health questionnaire (SHSQ-25) including 5 domains of 25 items [6]. Each subject was asked to rate a specific statement on a five-point Likert-type scale, based on how often they suffered various specific complaints in the preceding 3 months: (1) never or almost never, (2) occasionally, (3) often, (4) very often, and (5) always. The raw scores of 1 to 5 on the questionnaire were recoded as 0 to 4. SHS scores were calculated for each respondent by summing the ratings for the 25 items. The Cronbach’s α coefficient of the SHSQ-25 was 0.91, indicating good internal consistency.

We included classical risk factors, i.e., blood pressure, glucose, lipid levels, cortisol, and body mass index (BMI) in our analyses. Blood pressure was measured three times consecutively after 5 minutes rest, with each participant seated and using a standard mercury sphygmomanometer. The average of the second and third measurements was used to estimate the systolic and diastolic blood pressures. Overnight fasting blood specimens were obtained for the measurement of plasma glucose, serum lipids, and cortisol. Plasma glucose was measured using a modified hexokinase enzymatic method (Hitachi automatic clinical analyzer, model 7060, Japan). Concentrations of total cholesterol, highdensity lipoprotein (HDL) cholesterol, and triglycerides (TG) were assessed enzymatically with commercially available reagents. Lipid measurements were standardized according to the criteria of the Centers for Disease Control and Prevention-National Heart, Lung, and Blood Institute Lipid Standardization Program. Low-density lipoprotein (LDL) cholesterol was computed by the Friedewald formula using the equation (LDL = total cholesterol&[HDL + TG/5]) [8]. Fasting cortisol was analyzed by γ radioimmunoassay (RIA) counter (GC-911) in the Endocrinology Institute. The intra-assay and inter-assay coefficient of variations (CVs) of this assay were <5.5% and <7.5% respectively. The reference value was 50-280 ng/ml. Strict quality controls were applied throughout all these assays.

Body weight and height were measured twice during the interview. Weight was measured in light indoor clothing without shoes on electronic scales placed on a firm, level surface to the nearest 0.1 kg. Height was measured without shoes with a wall-mounted stadiometer to the nearest 0.1 cm. BMI was calculated as body weight (in kilograms) divided by height (in meters) squared.

Data on sociodemographic information and health-related behaviors were collected during interviews. These variables were used as confounders to control for potential confounding. Demographic variables included age, education, occupation, average monthly income, and marital status. The health-related behaviors include current smoking, alcohol use, and physical activity. Smoking status was dichotomized as current smoker (≥1 filter per day) and nonsmoker. Physical activity was assessed by asking to list the average hours of physical activity for each day of the week prior to the questionnaire.

Our analysis was restricted to 3,019 individuals who had completed the questionnaire and laboratory results. Their mean age was 40.6 years (SD 13.4) and 48.6% were women. The participants were divided into two groups by the median SHS score of 44: high SHS score group (SHS score ≥44) and low SHS score group (SHS <44). The mean SHS score among the SHS group was 55.73 ± 9.58 and 35.02 ± 6.51 among the control group, respectively. Table 1 presents the proportion of white collar workers and those with university/college degree among the high score group was significantly higher than that among the low-score group (P < 0.001). Gender and age distribution were also different between the two groups (P < 0.001). Since over half of the individuals aged over 50 years were excluded because of the results of their general medical examination, the number of this age group was significantly lower than the younger age groups. SHS was correlated with current smoking and physical inactivity (P < 0.001), whereas monthly income, marital status, and alcohol use were not different between groups.

**Table 1:** Characteristics of study sample

**Variables**	**SHS sore ≥ 44**	**SHS sore < 44**	**χ**^**2**^	***P***
	**n (%)**	**n (%)**		
Gender				
Female	806 (52.10)	660 (44.84)	15.933	<0.001
Male	741 (47.90)	812 (55.16)		
Age				
20-30 years	167 (10.80)	376 (25.54)	128.978	<0.001
31-40 years	606 (39.17)	575 (39.06)		
41-50 years	559 (36.13)	380 (25.82)		
51-60 years	215 (13.90)	141 (9.58)		
Highest education level				
Compulsory school (through grade 9)	72 (4.65)	171 (11.62)	185.420	<0.001
High school graduation	213 (13.77)	432 (29.35)		
University/college degree	1262 (81.58)	869 (59.04)		
Occupation				
White-collar worker	1431 (92.50)	986 (66.98)	307.665	<0.001
Blue-collar worker	116 (7.50)	486 (33.02)		
Monthly income (RMB)				
<2000	324 (20.94)	316 (21.47)	3.852	0.146
2001-5000	1012 (65.42)	990 (67.26)		
≥5000	211 (13.64)	166 (11.28)		
Marital status				
Single or divorced	196 (12.67)	168 (11.41)	1.123	0.289
Married	1351 (87.33)	1304 (88.59)		
Current smoking				
Yes	476 (30.77)	187 (12.70)	143.638	<0.001
No	1071 (69.23)	1285 (87.30)		
Alcohol use				
Every day	72 (4.65)	54 (3.67)	2.309	0.511
3-4 × a week	351 (22.69)	348 (23.64)		
1-2 × a week	988 (63.87)	948 (64.40)		
Never	136 (8.79)	122 (8.29)		
Physical activity				
≥ 5 hours	44 (2.84)	47 (3.19)	31.772	<0.001
3-4 hours	341 (22.04)	437 (29.69)		
1-2 hours	641 (41.44)	486 (33.02)		
< 1 hours	521 (33.68)	502 (34.10)		

Overall, the participants who gave higher SHS score also had a higher risk of cardiovascular disease than those with lower scores (Table 2). Compared to the low-score group, systolic and diastolic blood pressure, plasma glucose, total cholesterol, triglyceride levels, and BMI were significantly higher among the high score group (P < 0.001). Meanwhile HDL cholesterol levels were higher in low-score group than in high-score group (P > 0.05). Serum cortisol level was much higher among the high-score group than that among the low-score group (204.31 versus 161.33 ng/ml, P < 0.001). The ranges of cortisol in high-score and low-score group were 122.64-324.17 and 107.12-221.59 ng/ml, respectively. A significant linear correlation between SHS sore and serum cortisol was evident (r = 0.381, P < 0.001).

**Table 2:** Comparision of the cardiovascular risk factors between high and low SHS score group

	**SHS score high**	**SHS score low**	**t**	***P *value**^**a**^
	**Mean** + **Std**.	**Mean** + **Std**.		
SBP (mmHg)	119.43 ± 13.27	115.31 ± 13.19	8.573	<0.001
DBP (mmHg)	77.57 ± 7.38	75.38 ± 7.89	7.880	<0.001
GLU (mmol/L)	5.23 ± 0.57	5.17 ± 0.55	2.941	<0.001
TCH (mmol/L)	4.48 ± 0.76	4.32 ± 0.78	5.708	<0.001
TG (mmol/L)	1.17 ± 0.58	1.08 ± 0.46	4.709	<0.001
HDLC (mmol/L)	1.32 ± 0.32	1.36 ± 0.36	−3.230	<0.001
LDLC (mmol/L)	2.82 ± 0.70	2.78 ± 0.71	1.558	0.119
COR (ng/ml)	204.31 ± 40.06	161.33 ± 27.83	34.076	<0.001
BMI (kg/m^2^)	23.24 ± 3.76	22.01 ± 3.52	9.268	<0.001

SBP, systolic blood pressure; DBP, diastolic blood pressure; GLU, plasma glucose; TCH, total cholesterol; TG, triglyceride; HDLC, high-density lipoprotein cholesterol; LDLC, low-density lipoprotein cholesterol; COR, serum cortisol.

We further used linear two-level model to analyze the association of SHS with the cardiovascular risk factors, with participants’ characteristics showing difference between the high and low SHS score groups as controlling variables and sex as a stratifying variable. In the models, SHS score was treated as a continuous variable.

After adjusted for age, education background, occupation, smoking, and physical activity, the diastolic blood pressure, plasma glucose, total cholesterol, and serum cortisol were found to significantly predict SHS score (P < 0.05) among man (Table 3). HDL cholesterol level was negatively and significantly associated with SHS score (P < 0.05). In the fully adjusted multilevel analysis, no significant association was observed between triglyceride, LDL cholesterol, BMI, and SHS score (P > 0.05). As the major risk factors for most chronic diseases, smoking and lack of physical activity also significantly associated with SHS score among men (P < 0.05).

**Table 3:** Multilevel estimates for SHS score in relation to cardiovascular risk factors among male participants

	**Estimate(b)**	**SE**	***P *value**
Systolic blood pressure	0.601	0.211	0.004
Diastolic blood pressure	0.486	0.230	0.035
Plasma glucose	0.636	0.302	0.035
Total cholesterol	1.003	0.333	0.003
Triglyceride	0.477	0.293	0.104
HDL cholesterol	−0.986	0.400	0.014
LDL cholesterol	0.160	0.116	0.168
Serum cortisol	0.231	0.004	<0.001
Body mass index	0.180	0.214	0.400
Level 2 (person) intercept variance (SE)		6.903 (1.369)	
Level 2 (company) intercept variance (SE)		3.418 (1.192)	

The results of parameter estimates and standard errors from the two-level model among women were similar to those among men, with the exception of plasma glucose and HDL cholesterol (Table 4). After adjusted for age, education background, occupation, smoking, and physical activity, the diastolic blood pressure, total cholesterol, triglyceride, HDL cholesterol, and serum cortisol were found to significantly predict SHS score among woman (P < 0.05). No significant association was observed between plasma glucose, LDL cholesterol, BMI, and SHS score (P > 0.05). Smoking and lack of physical activity also significantly associated with SHS score among women (P < 0.05).

**Table 4:** Multilevel estimates for SHS score in relation to cardiovascular risk factors among female participants

	**Estimate(b)**	**SE**	***P *value**
Systolic blood pressure	0.388	0.181	0.032
Diastolic blood pressure	0.751	0.280	0.007
Plasma glucose	0.151	0.116	0.193
Total cholesterol	1.353	0.423	0.001
Triglyceride	1.245	0.407	0.002
HDL cholesterol	−1.516	0.669	0.024
LDL cholesterol	0.420	0.365	0.250
Serum cortisol	0.225	0.005	<0.001
Body mass index	0.250	0.197	0.205
Level 2 (person) intercept variance (SE)		4.152 (1.530)	
Level 2 (company) intercept variance (SE)		2.414 (1.116)	

We got similar results when we analyzed the relationship between total sore of 5 questionnaire items about cardiovascular domain and cardiovascular risks among man and woman.

With SHSQ-25, a large cross-sectional study was carried out to address the relationship of self-rated ill health with classical cardiovascular risk factors in the primary prevention of cardiovascular disease. This study is conducted in a representative sample of workers in urban Beijing. The response rate was high (88.7%), which therefore limited the potential selection bias. We found correlation between SHS and systolic blood pressure, diastolic blood pressure, plasma glucose, total cholesterol, and HDL cholesterol among men and correlation between SHS and systolic blood pressure, diastolic blood pressure, total cholesterol, triglyceride, and HDL cholesterol among women.

In our study, a newly created instrument, SHSQ-25, was used for measurement of SHS. The SHSQ-25 is a self-rated questionnaire of perceived health complaints [6,7]. SHS is more prevalent in women than in men and more in white-collar workers than in blue-collar workers. In addition, the prevalence of SHS increases with age. This trend is consistent with the prevalence of metabolic syndrome and cardiovascular disease in urban China. The similarity could partially be accounted by sharing common risk factors. Significant higher level of serum cortisol among high SHS score group compared to low-score group (t = 34.076, P < 0.001) and significant linear correlation between SHS score and serum cortisol (r = 0.381, P < 0.001) strengthen the evidence that stress is an important related factor for SHS. With the rapid economic progress across China, employees have been becoming more exposed to stressful situations such as excessive workload, competition, and perceived loneliness [9].

Continuous psychosocial stress seems to be a part of the everyday life in Chinese, especially among whitecollar workers [10]. Endocrine measures of stress and self-rated health (SRH) were also proved in a longitudinal study: poorer SRH at each point in time was associated with higher levels of serum cortisol and prolactin [11]. SRH may capture subclinical or undiagnosed disease [12]. In addition, the measures of perceived health modified the effects of biomedical risk factors both in the prediction of myocardial infarction and stroke [13,14]. A possible pathway for observed effects is that SRH reflects the presence or absence of psychosocial risk factors or resources [12]. Psychological stress can affect health not only directly through neuroendocrine responses, but also indirectly through changes in health behaviors [13]. Current smoking was significantly more common in individuals giving higher SHS score. They also reported significantly less physical activity.

In China, chronic noncommunicable diseases accounts for about 80% of deaths and 70% of disability-adjusted life-years [15,16]. As a developing country with huge population, it is imperative that an economical and valid instrument is developed for screening major chronic diseases. The SHSQ-25 is short and easy to be completed, and therefore, suitable for use in general population and primary care service [6,7]. In many developed counties, much attention has been paid on perceived poor health “somatization” and “medically unexplained symptoms” in community and primary care system [17,18]. Somatic symptom is one of the main reasons for patients seeking health care [19,20]. SHS cannot be fully understood from the conventional disease-oriented biomedical point of view. Instead, it requires a holistic biopsychosocial perspective in which complaints are viewed as the result of complex interactions of physiology, psychology, and social environment. Primary care providers must be able to detect and manage SHS. The SHSQ-25 is a valid instrument for such purpose.

Effective intervention on SHS may be a cost-effective way for preventing chronic diseases. One critical premise of disease-related biomarkers (genomics, proteomics, glycomics and metabolomics) is also the key for the early diagnosis and prevention and management of suboptimal health [21,22].

## Conclusion

The final questionnaire congregated into a score (SHSQ-25) which could significantly distinguish among several abnormal conditions and could be used as a translational medicine instrument for health measuring in the general population.

## Competing interests

The authors declare that they have no competing interests.

## Authors' contributions

All of the authors helped with the design of the study, and as well as the analysis or interpretation of the data. All authors assisted in either drafting the manuscript or revising it. All authors read and approved the final manuscript.

## Supplementary Material

Additional file 1 Sub-health Status Questionnaire. Click here for file
