# Supplementary material for: Suboptimal health: a new health dimension for translational medicine
Source: Clin Transl Med. 2012 Nov 14;1:28. doi: 10.1186/2001-1326-1-28 (PMC3561061; doi:10.1186/2001-1326-1-28)
Supplement: Additional file 1 — Sub-health Status Questionnaire. [file 2001-1326-1-28-S1.docx]

*The following questions ask some events about your health during the last 3 months. Answer*

*very question by making the appropriate box with an 'x'. You may choose from one of the*

*following answers:*
